# 2-Heptylcyclopropane-1-Carboxylic Acid Disperses and Inhibits Bacterial Biofilms

**DOI:** 10.3389/fmicb.2021.645180

**Published:** 2021-06-09

**Authors:** Zoe L. Harrison, Rukhsana Awais, Michael Harris, Babatunde Raji, Brian C. Hoffman, Daniel L. Baker, Jessica Amber Jennings

**Affiliations:** ^1^Department of Biomedical Engineering, University of Memphis, Memphis, TN, United States; ^2^Department of Chemistry, University of Memphis, Memphis, TN, United States

**Keywords:** biofilm, dispersal, *Staphylococcus aureus*, *Pseudomonas aeruginosa*, diffusible signaling factors, 2-decenoic acid, anti-biofilm agents

## Abstract

Fatty-acid signaling molecules can inhibit biofilm formation, signal dispersal events, and revert dormant cells within biofilms to a metabolically active state. We synthesized 2-heptylcyclopropane-1-carboxylic acid (2CP), an analog of *cis*-2-decenoic acid (C2DA), which contains a cyclopropanated bond that may lock the signaling factor in an active state and prevent isomerization to its least active *trans*-configuration (T2DA). 2CP was compared to C2DA and T2DA for ability to disperse biofilms formed by *Staphylococcus aureus* and *Pseudomonas aeruginosa*. 2CP at 125 μg/ml dispersed approximately 100% of *S. aureus* cells compared to 25% for C2DA; both 2CP and C2DA had significantly less *S. aureus* biofilm remaining compared to T2DA, which achieved no significant dispersal. 2CP at 125 μg/ml dispersed approximately 60% of *P. aeruginosa* biofilms, whereas C2DA and T2DA at the same concentration dispersed 40%. When combined with antibiotics tobramycin, tetracycline, or levofloxacin, 2CP decreased the minimum concentration required for biofilm inhibition and eradication, demonstrating synergistic and additive responses for certain combinations. Furthermore, 2CP supported fibroblast viability above 80% for concentrations below 1 mg/ml. This study demonstrates that 2CP shows similar or improved efficacy in biofilm dispersion, inhibition, and eradication compared to C2DA and T2DA and thus may be promising for use in preventing infection for healthcare applications.

## Introduction

A biofilm is an aggregate of microbial cells that attaches to various surfaces, including tissue and medical implants (Stoodley et al., 2013; Saeed et al., 2019). Almost 80% of human infections are caused by the development of biofilms by pathogenic bacterial strains such as *Staphylococcus aureus*, *Staphylococcus epidermis*, *Pseudomonas aeruginosa*, and *Escherichia coli* (Brady et al., 2006). The attachment of these and other bacterial strains to implant or tissue surfaces to form biofilms poses a particular risk for recurring infections, as the cells within a biofilm are resistant to both antibiotics and immune cell clearance (Bauer and Grosso, 2013). The recalcitrance of biofilms to treatment is multifactorial, owing in part to secretion of exopolymeric substances (EPS) and alteration of the bacterial metabolic state (Flemming et al., 2016). EPS secretion enhances bacterial attachment, resistance to mechanical stresses, and facilitation of nutrient transport and may limit diffusion of antimicrobials and immune cell penetration (Molin and Tolker-Nielsen, 2003; Czaczyk and Myszka, 2007; Flemming, 2016). Immobilized subgroups of bacterial cells within the biofilm have decreased metabolic activity and can increase the minimum inhibitory concentration of antibiotics up to 1,000 times higher for biofilms compared to planktonic bacteria (Keren et al., 2004). These and other complexities associated with biofilm infections have led to extensive research of their mechanism of action and potential novel treatments.

Although current clinical treatments for biofilm infections typically include debridement of infected tissue and prescription of high-dose systemic antibiotics, there are a number of non-antibiotic methods currently being studied for their potential to prevent and treat biofilms. Some of these therapies include natural-derived molecules targeting specific aspects of the biofilm life cycle. Enzymes such as glycoside hydrolases and proteases disperse biofilm bacteria by degrading the EPS (Jennings et al., 2015). Antimicrobial peptides (AMPs) typically have cationic amphipathic structures that can disrupt bacterial cell membranes to inhibit biofilms (Batoni et al., 2016). Hydrophobic D-amino acids are secreted by bacteria prior to dispersal of biofilms and have been shown to inhibit biofilm growth *in vitro* and *in vivo*, although their mechanism of action remains unclear (Hochbaum et al., 2011). Some types of bacteria also secrete biosurfactant molecules called rhamnolipids (Nickzad and Déziel, 2014), which inhibit bacterial attachment to surfaces and trigger degradation of the EPS during dispersal. Sugar alcohols like mannitol and erythritol have been shown to stimulate metabolism of bacteria that causes awakening of dormant cells and increased susceptibility to antibiotics (Pace et al., 2019). AMPs and the sugar alcohol mannitol have been shown to eradicate both *P. aeruginosa* and *S. aureus* infections when combined with typical concentrations of antibiotics (Barraud et al., 2013; Dosler and Karaaslan, 2014; de Breij et al., 2016; Pace et al., 2019), although neither of these methods have been attempted clinically.

A class of medium-chain fatty-acid molecules has been shown to induce dispersion of preformed biofilms and inhibit biofilm formation (Davies and Marques, 2009; Ryan and Dow, 2011; Jennings et al., 2012). These specific medium-chain fatty acids are members of a family of diffusible signal factors (DSF) in bacteria. DSF are secreted by bacteria to function in cell–cell communication or quorum sensing (Solano et al., 2014). One well-studied DSF molecule is *cis*-2-decenoic acid (C2DA), which is released by *P. aeruginosa* and has been shown to inhibit biofilm formation and to disperse established biofilms of multiple strains (Davies and Marques, 2009). However, other studies have shown difficulty in reproducing these results, with less than 10% dispersion of *S. aureus* and *Acinetobacter baumannii* strains at 400 μM concentration (Su et al., 2011). It has been observed that some of these fatty-acid signaling factors revert dormant bacterial cells to a metabolically active state (Marques et al., 2014), which in combination with antimicrobials could decrease bacterial viability (Allison et al., 2011; Rahmani-Badi et al., 2014; Masters et al., 2016; Saeed et al., 2019). Moreover, these compounds have cross-kingdom efficacy in that they have been shown to inhibit and disperse biofilms formed by multiple types of microorganisms, including gram-positive bacteria, gram-negative bacteria, and fungi (Wang et al., 2004). C2DA was also shown to inhibit *S. aureus* growth at a concentration of 500 μg/ml and to prevent biofilm formation at a concentration of 125 μg/ml, with neither concentration showing cytotoxic effects in fibroblasts (Jennings et al., 2012). Dispersal and inhibition effects may be mediated by activation of gene pathways that control motility, metabolism, and persistence (Amari et al., 2013; Rahmani-Badi et al., 2015b). While mechanisms of action remain unclear (Jennings et al., 2012), recent work shows that the *cis*-conformation of this molecule increases membrane permeability and could allow for entry of more small-molecule antibiotics into the cells as compared to the less active *trans*-isomer (Masters et al., 2016).

Activity of DSF molecules appears to be dependent on the conformation of atoms around the point of unsaturation at carbon 2. *cis*-Alkenes may isomerize to *trans*- when exposed to light, elevated temperature, or radiation (Dugave and Demange, 2003). These conditions, one or more of which may be used in the fabrication and/or sterilization of medical devices or therapeutics, can lead to isomerization of the alkene portion of the fatty-acid DSF (Tipnis and Burgess, 2018). In this study, we have developed chemical routes for synthesis of a cyclopropanated analog, 2-heptylcyclopropane-1-carboxylic acid (2CP), that locks this molecule into a *cis*-like configuration. This configuration eliminates the potential for *cis*/*trans*-isomerization and oxidative degradation that is possible for C2DA when exposed to light, heat, and radiation. We evaluated the hypothesis that 2CP disperses and inhibits biofilm using *in vitro* assays of *S. aureus* and *P. aeruginosa* biofilm dispersion. These were chosen as representative specimens as *S. aureus* is a major pathogen in bone infection, and *P. aeruginosa* is prevalent in soft tissue infection; both strains selected for this study are clinically derived. We further evaluated the combination of 2CP with antibiotics to identify synergistic and additive responses through minimum biofilm inhibitory concentration (MBIC) and minimum biofilm eradication concentration (MBEC) assays compared to C2DA and T2DA. We compared compatibility with mammalian cells at concentrations active against biofilms for all three 2-decenoic acid analogs and compared preliminary stability of 2CP to C2DA in an accelerated ultraviolet light-driven degradation scenario.

## Materials and Methods

### Synthesis of C2DA and 2CP

A common synthetic approach using a single starting material was used to generate C2DA *via* Jones oxidation and then Lindlar reduction, and 2CP *via* Lindlar reduction, Simmons–Smith cyclopropanation, and finally Jones oxidation (Supplementary Figure 1). ^1^HNMR was performed to determine whether 2CP had been synthesized with a “*cis*-like” conformation (Supplementary Figures 2, 3). T2DA was purchased from Cayman Chemical and was used without purification.

### Dispersion

Dispersion assays were performed to compare the dispersion activity of each analog against 7-day biofilms. 2CP (stabilized version of C2DA) was compared with C2DA (active, positive control) and T2DA (inactive/active, negative control). Each well of a 96-well plate was seeded with 150 μl of bacterial culture (*S. aureus*, UAMS-1, or *P. aeruginosa*, PA-ATCC 27317) and incubated for 7 days at 37°C to result in biofilms. Media [Tryptic Soy Broth (TSB)] was changed after careful aspiration every 24 h. On day 7, each well was again carefully aspirated and then received 195 μl of TSB and 5 μl of fatty-acid stock ranging in concentration from 0 to 500 μg/ml. Fatty-acid concentrations were made in absolute ethanol; thus, the final ethanol concentration in wells was 2.5%. The plates were incubated for 24 h at 37°C, then turbidity was measured at 540 nm. Turbidity readings were performed using a Biotek Synergy^TM^ H1 microplate reader, with increased turbidity readings indicating a higher number of viable bacterial cells. Afterward, the media was aspirated and the plate was washed three times using phosphate-buffered saline (PBS). The relative percentage of attached cells as compared to non-treated controls were measured using BacTiter-Glo^TM^ (Promega) Luciferase assay for ATP production, with higher percentages indicating a higher quantity of attached biofilm cells.

### Inhibition

Synergy assays were performed to determine the effects of 2CP when used in combination with various antimicrobials against gram-positive *S. aureus* and gram-negative *P. aeruginosa*. Tobramycin (MP Biomedicals, Irvine, CA, United States), tetracycline (MP Biomedicals, Irvine, CA, United States), and levofloxacin (Alfa Aesar, Tewksbury, MA, United States) were chosen for evaluation based on prior work, demonstrating their synergistic effects with C2DA (Masters et al., 2016). A checkerboard assay was prepared in 96-well plates with increasing antibiotic concentration on the horizontal axis and increasing 2CP concentration on the vertical axis. Tested concentrations of 2CP, C2DA, and T2DA ranged from 0 to 4,000 μg/ml. Antibiotic concentrations tested ranged from 0 to 2,500 μg/ml. Each plate was then inoculated with *S. aureus* (UAMS-1) or *P. aeruginosa* (PA-ATCC 27317) overnight growths for a final dilution of 1 in 50 or 1 in 200, respectively, and incubated overnight. Inhibition was defined as a lack of visible growth after a 24-h incubation period.

The fractional inhibitory concentration index (FICI) was used to determine synergistic, additive, or antagonistic responses between the three antibiotics and three fatty acids. To calculate the FICI, the MIC of the antibiotic when combined with fatty acids was divided by the MIC of the antibiotic alone and added to the MIC of fatty acid when combined with antibiotics and divided by the MIC of fatty acid when applied alone (Equation 1). FICI values less than or equal to 0.5 indicate synergy, values between 0.5 and 1 indicate additivity, values between 1 and 2 indicate indifference, and values above 2 indicate antagonism (Equation 1) (European Committee for Antimicrobial Susceptibility Testing (EUCAST) of the European Society of Clinical Microbiology and Infectious Dieases (ESCMID), 2000).

$$F\,I\,C\,I=\frac{M\,I\,C_{A\,B\,X\,\left(C\right)}}{M\,I\,C_{A\,B\,X\,\left(S\right)}}+\frac{M\,I\,C_{F\,A\,\left(C\right)}}{M\,I\,C_{F\,A\,\left(S\right)}}$$

**Equation 1**. FICI calculation using the concentrations of antibiotics (ABX) and fatty acids (FA) alone (S) and in combination (C).

### Eradication

Bacteria (*S. aureus*, UAMS-1, or *P. aeruginosa*, PA-ATCC 27317) were grown overnight in TSB by incubation at 37°C. One milliliter of bacterial solution was diluted in 9 ml of TSB. An amount of 150 μl of the bacterial stock solution was seeded into each well of a 96-well MBEC^TM^ Biofilm Inoculator plate, followed by overnight incubation at 37°C to form the biofilm. The next day, the medium from each well was carefully aspirated, allowing the biofilm to remain both on the pegs of the top plate and the bottom of the wells. Stocks of tobramycin, tetracycline, and levofloxacin as well as varying concentrations of C2DA, T2DA, and 2CP were added to the wells. Tested concentrations of 2CP, C2DA, and T2DA ranged from 0 to 4,000 μg/ml. Antibiotic concentrations tested ranged from 0 to 2,500 μg/ml. These plates were incubated at 37°C for 24 h. The next day, the pegged lids were removed and added to new plates containing 150 μl of sterile TSB in each well. Plates were sonicated for 5 min at 40 kHz (Fisher Scientific Ultrasonic Bath, 9.5 l) to remove the viable bacteria attached to the peg surface and then incubated for 24 h. Turbidity was measured by reading absorbance at 540 nm for bacterial growth in the presence of fixed concentrations of the antibiotics with varying concentrations of 2CP, C2DA, and T2DA. MBECs were determined by the lowest concentration that had no turbid growth after treatment and sonication.

The fractional biofilm eradication concentration (FBEC) index was used to determine synergistic, additive, or antagonistic responses between the three antibiotics and three fatty acids. The FBEC index was determined in an identical method to the FICI: the MBEC of the antibiotic when combined with fatty acids was divided by the MBEC of the antibiotic alone and added to the MBEC of fatty acid when combined with antibiotics and divided by the MBEC of fatty acid when applied alone (Equation 2). FBEC index values less than or equal to 0.5 indicate synergy, values between 0.5 and 1 indicate additivity, values between 1 and 2 indicate indifference, and values above 2 indicate antagonism.

$$F\,B\,E\,C=\frac{M\,B\,E\,C_{A\,B\,X\,\left(C\right)}}{M\,B\,E\,C_{A\,B\,X\,\left(S\right)}}+\frac{M\,B\,E\,C_{F\,A\,\left(C\right)}}{M\,B\,E\,C_{F\,A\,\left(S\right)}}$$

**Equation 2**. FBEC index calculation using the concentrations of antibiotics (ABX) and fatty acids (FA) alone (S) and in combination (C).

### Cytocompatibility

NIH-3T3 fibroblasts were seeded (1 × 10^4^ cells/cm^2^) in 24-well plates in Dulbecco’s modified Eagle’s medium (DMEM) supplemented with 10% fetal bovine serum and 100 μg/ml Normocin (Invivogen, San Diego, CA, United States) for 24 h at 37°C and 5% CO_2_. 2CP, C2DA, and T2DA were added in varying concentrations from 0 to 1,000 μg/ml in ethanol for a final ethanol concentration of 2.5%. Controls with no ethanol addition were also used to normalize percentage viability. Plates were incubated for 24 h, after which cell viability was measured using CellTiter-Glo^®^ (Promega, Madison, WI, United States).

### Stability of C2DA and 2CP

Oxidative degradation and isomerization of C2DA and 2CP were assessed using negative-ion LC-MS to quantify the loss of each parent DSF following exposure to the photoinitiator Irgacure 2959 (Sigma-Aldrich, St. Louis, MO, United States). Aqueous solutions (10 ml total volume, 10% final methanol concentration) of C2DA and 2CP (200 ng/μl) were prepared from methanolic stocks. Irgacure was also prepared as a methanolic stock with final concentrations varying from 0, 0.05, and 0.1% (w/v). Solutions of C2DA or 2CP with and without the initiator were then exposed to UV at 365 nm for up to 4 h using a Spectrolinker XL-1500 UV crosslinker (Westbury, NY, United States). Triplicate samples (200 μl) were taken at timepoints between 0 and 240 min and were extracted by the method of Bligh and Dyer. The chloroform extracts were then dried and resuspended in 4 ml of HPLC-grade water. Samples (10 μl) were analyzed *via* negative-ion LC-ESI-MS, using a Thermo Fisher Hypersil BDS C18 column and an isocratic mobile phase of 55: 45 (v/v) methanol: aqueous ammonium hydroxide (10 μM). Peak areas for molecular anions of C2DA and 2CP were quantified using calibration curves using decanoic acid as an internal standard. The concentration of each sample was calculated, and the change in concentration of 2CP and C2DA over time was tracked.

### Statistical Analysis

Statistical analysis was performed using SigmaPlot (Systat Software Inc., San Jose, CA, United States) and GraphPad Prism 7.2 software (GraphPad Software Incorporation, La Jolla, CA, United States). Data was assessed first by performing the Shapiro–Wilk normality test, followed by the Brown–Forsythe equal variance test. If both passed, data was further analyzed with one-way analysis of variance (ANOVA) followed by Holm–Sidak *post-hoc* analysis to detect significant between experimental groups (α = 0.05). If normality and equal variance were not passed, data was analyzed using Kruskal–Wallis ANOVA on ranks, followed by the Tukey *post-hoc* test.

## Results

### Dispersion

**FIGURE 1:**
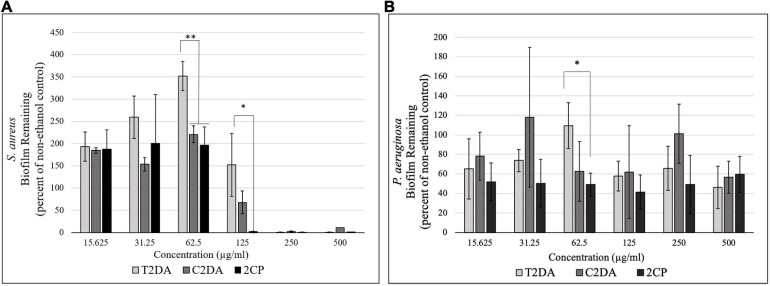
Graph showing the percentage of **(A)** *S. aureus* and **(B)** *P. aeruginosa* biofilm remaining after 1 h of exposure to T2DA, C2DA, and 2CP. Asterisks represent significantly less biofilm remaining compared to T2DA (^∗^*p* < 0.05, ^∗∗^*p* < 0.01), detected by one-way ANOVA with the Holm–Sidak *post-hoc* test. Data represent mean ± standard deviation.

2CP at 125 μg/ml dispersed approximately 100% of *S. aureus* biofilms grown for 24 h, compared to wells treated with C2DA which had 75% of biofilm remaining compared to untreated controls; T2DA-treated biofilms did not show any dispersion at this concentration. At concentrations of 62.5 μg/ml and above, both 2CP and C2DA had significantly less *S. aureus* biofilm remaining in wells compared to wells treated with T2DA (*p* < 0.05), which showed no significant dispersal (Figure 1A) and was statistically similar to the non-ethanol control group. 2CP concentrations ranging from 15.625 to 500 μg/ml dispersed approximately 40–60% of *P. aeruginosa* biofilm, which was comparable to the dispersion activity of C2DA and T2DA at the same concentrations. Statistical differences between *P. aeruginosa* dispersion between fatty-acid analogs were only observed between 2CP and T2DA at only the 62.5-μg/ml concentration (*p* > 0.05) (Figure 1B).

### Inhibition

The fatty-acid 2CP inhibited bacterial growth at high concentrations, inhibiting *S. aureus* at 1 mg/ml and *P. aeruginosa* growth at 4 mg/ml. Various concentrations of 2CP were shown to effectively reduce the MIC of tested antibiotics against both strains by at least 50%, with the exception of only tetracycline and *S. aureus*. Tobramycin was found to have additive effects with 2CP, C2DA, and T2DA against *S. aureus* growth, although 2CP had the lowest FICI score of 0.31, compared to 1.0 for C2DA and T2DA (Table 1). Tetracycline and levofloxacin were found to have additive effects with all three fatty acids against *S. aureus* growth. Similarly, tobramycin and tetracycline were additive with all three fatty acids for *P. aeruginosa* inhibition (Table 2). Although additive effects were found for levofloxacin combined with 2CP against *P. aeruginosa*, indifference or no effect was observed when combined with C2DA or T2DA.

**TABLE 1:** *S. aureus* fractional inhibitory concentration index values.

	**MBIC for 2CP (alone) (μg/ml)**	**MBIC for 2CP (combined) (μg/ml)**	**MBIC for antibiotic (alone) (μg/ml)**	**MBIC for antibiotic (combined) (μg/ml)**	**FICI**	**Interpretation**
***S. aureus***						
Tobramycin	1000.0	31	4.0	2.0	0.531	Additive
Tetracycline	1000.0	63	1.0	1.0	1.06	Indifferent
Levofloxacin	1000.0	500	1.0	0.3	0.8	Additive

	**MBIC for C2DA (alone) (μg/ml)**	**MBIC for C2DA (combined) (μg/ml)**	**MBIC for antibiotic (alone) (μg/ml)**	**MBIC for antibiotic (combined) (μg/ml)**	**FICI**	**Interpretation**

Tobramycin	1000.0	500	4.0	2.0	1.0	Additive
Tetracycline	1000.0	250	1.0	0.5	0.75	Additive
Levofloxacin	1000.0	250	1.0	0.5	0.75	Additive

	**MBIC for T2DA (alone) (μg/ml)**	**MBIC for T2DA (combined) (μg/ml)**	**MBIC for antibiotic (alone) (μg/ml)**	**MBIC for antibiotic (combined) (μg/ml)**	**FICI**	**Interpretation**

Tobramycin	1000.0	500	4.0	2.0	1.0	Additive
Tetracycline	1000.0	250	1.0	0.5	0.75	Additive
Levofloxacin	1000.0	250	1.0	0.5	0.75	Additive

*Minimum biofilm inhibitory concentration (MBIC) for 2CP, C2DA, and T2DA against S. aureus when treated alone or combined with tobramycin, tetracycline, or levofloxacin. MBIC of each antibiotic against S. aureus alone is also included. These values were used in Equation 1 to determine the fractional inhibitory concentration index for each fatty acid/antibiotic combination. FICI values less than or equal to 0.5 indicate synergy, values between 0.5 and 1 indicate additivity, values between 1 and 2 indicate indifference, and values above 2 indicate antagonism.*

**TABLE 2:** *P. aeruginosa* fractional inhibitory concentration index values.

	**MBIC for 2CP (alone) (μg/ml)**	**MBIC for 2CP (combined) (μg/ml)**	**MBIC for antibiotic (alone) (μg/ml)**	**MBIC for antibiotic (combined) (μg/ml)**	**FICI**	**Interpretation**
***P. aeruginosa***						
Tobramycin	4,000	31	0.5	0.25	0.508	Additive
Tetracycline	4,000	1,000	32	16	0.75	Additive
Levofloxacin	4,000	1,000	4.0	2	0.75	Additive

	**MBIC for C2DA (alone) (μg/ml)**	**MBIC for C2DA (combined) (μg/ml)**	**MBIC for antibiotic (alone) (μg/ml)**	**MBIC for antibiotic (combined) (μg/ml)**	**FICI**	**Interpretation**

Tobramycin	4,000	125	0.5	0.25	0.531	Additive
Tetracycline	4,000	500	32	16	0.625	Additive
Levofloxacin	4,000	4,000	4.0	4.0	2.0	Indifferent

	**MBIC for T2DA (alone) (μg/ml)**	**MBIC for T2DA (combined) (μg/ml)**	**MBIC for antibiotic (alone) (μg/ml)**	**MBIC for antibiotic (combined) (μg/ml)**	**FICI**	**Interpretation**

Tobramycin	4,000	125	0.5	0.3	0.631	Additive
Tetracycline	4,000	1,000	32	16	0.75	Additive
Levofloxacin	4,000	4,000	4.0	4.0	2.0	Indifferent

*Minimum biofilm inhibitory concentration (MBIC) for 2CP, C2DA, and T2DA against P. aeruginosa when treated alone or combined with tobramycin, tetracycline, or levofloxacin. MBIC of each antibiotic against P. aeruginosa alone is also included. These values were used in Equation 1 to determine the fractional inhibitory concentration index for each fatty acid/antibiotic combination. FICI values less than or equal to 0.5 indicate synergy, values between 0.5 and 1 indicate additivity, values between 1 and 2 indicate indifference, and values above 2 indicate antagonism.*

### Eradication

The fatty-acid 2CP was not capable of eradicating *P. aeruginosa* biofilm when applied alone but at the concentration of 1,000 μg/ml was sufficient to eradicate *S. aureus*. Various concentrations of 2CP were shown to effectively reduce the MBEC of tobramycin, tetracycline, and levofloxacin against both strains by at least 50%. Tobramycin and tetracycline were found to have additive effects with all three fatty-acid analogs against *S. aureus* growth, although combining 2CP or C2DA with levofloxacin it had synergistic effects in eradication of biofilm (Table 3). Tobramycin and levofloxacin were synergistic with 2CP for eradication of *P. aeruginosa* (Table 4). Tetracycline did not result in eradication of *P. aeruginosa* biofilm at the highest concentration studied, which could not be increased in these assays due to solubility limitations.

**TABLE 3:** *S. aureus* FBEC index values.

	**MBEC for 2CP (alone) (μg/ml)**	**MBEC for 2CP (combined) (μg/ml)**	**MBEC for antibiotic (alone) (μg/ml)**	**MBEC for antibiotic (combined) (μg/ml)**	**FBEC**	**Interpretation**
***S. aureus***						
Tobramycin	1,000	31.25	2.5	1.25	0.531	Additive
Tetracycline	1,000	125	1.25	0.625	0.625	Additive
Levofloxacin	1,000	250	1	0.25	0.5	Synergistic

	**MBEC for C2DA (alone) (μg/ml)**	**MBEC for C2DA (combined) (μg/ml)**	**MBEC for antibiotic (alone) (μg/ml)**	**MBEC for antibiotic (combined) (μg/ml)**	**FBEC**	**Interpretation**

Tobramycin	1,000	125	2.5	1.25	0.625	Additive
Tetracycline	1,000	500	1	0.25	0.75	Additive
Levofloxacin	1,000	125	1	0.25	0.375	Synergistic

	**MBEC for T2DA (alone) (μg/ml)**	**MBEC for T2DA (combined) (μg/ml)**	**MBEC for antibiotic (alone) (μg/ml)**	**MBEC for antibiotic (combined) (μg/ml)**	**FBEC**	**Interpretation**

Tobramycin	1,000	125	2.5	1.25	0.625	Additive
Tetracycline	1,000	500	1	0.25	0.75	Additive
Levofloxacin	1,000	500	1	0.25	0.75	Additive

*Minimum biofilm eradication concentration (MBEC) for 2CP, C2DA, and T2DA against S. aureus biofilm when treated alone or combined with tobramycin, tetracycline, or levofloxacin. MBEC of each antibiotic against S. aureus alone is also included. These values were used in Equation 2 to determine the fractional inhibitory concentration index for each fatty acid/antibiotic combination. FBEC index values less than or equal to 0.5 indicate synergy, values between 0.5 and 1 indicate additivity, values between 1 and 2 indicate indifference, and values above 2 indicate antagonism.*

**TABLE 4:** *P. aeruginosa* fractional eradication concentration index values.

	**MBEC for 2CP (alone) (μg/ml)**	**MBEC for 2CP (combined) (μg/ml)**	**MBEC for antibiotic (alone) (μg/ml)**	**MBEC for antibiotic (combined) (μg/ml)**	**FBEC**	**Interpretation**
***P. aeruginosa***						
Tobramycin	>4,000	250	500	150	0.363	Synergistic
Tetracycline	>4,000	ND	ND	ND	ND	ND
Levofloxacin	>4,000	250	312	78	0.313	Synergistic

	**MBEC for C2DA (alone) (μg/ml)**	**MBEC for C2DA (combined) (μg/ml)**	**MBEC for antibiotic (alone) (μg/ml)**	**MBEC for antibiotic (combined) (μg/ml)**	**FBEC**	**Interpretation**

Tobramycin	>4,000	250	2500	625	0.313	Synergistic
Tetracycline	>4,000	ND	ND	ND	ND	ND
Levofloxacin	>4,000	500	312	78	0.375	Synergistic

	**MBEC for T2DA (alone) (μg/ml)**	**MBEC for T2DA (combined) (μg/ml)**	**MBEC for antibiotic (alone) (μg/ml)**	**MBEC for antibiotic (combined) (μg/ml)**	**FBEC**	**Interpretation**

Tobramycin	>4,000	250	2,500	625	0.313	Synergistic
Tetracycline	>4,000	ND	ND	ND	ND	ND
Levofloxacin	>4,000	500	312	78	0.375	Synergistic

*Minimum biofilm eradication concentration (MBEC) for 2CP, C2DA, and T2DA against P. aeruginosa biofilm when treated alone or combined with tobramycin, tetracycline, or levofloxacin. MBEC of each antibiotic against P. aeruginosa alone is also included. These values were used in Equation 2 to determine the fractional inhibitory concentration index for each fatty acid/antibiotic combination. FBEC index values less than or equal to 0.5 indicate synergy, values between 0.5 and 1 indicate additivity, values between 1 and 2 indicate indifference, and values above 2 indicate antagonism.*

### Cytocompatibility

**FIGURE 2:**
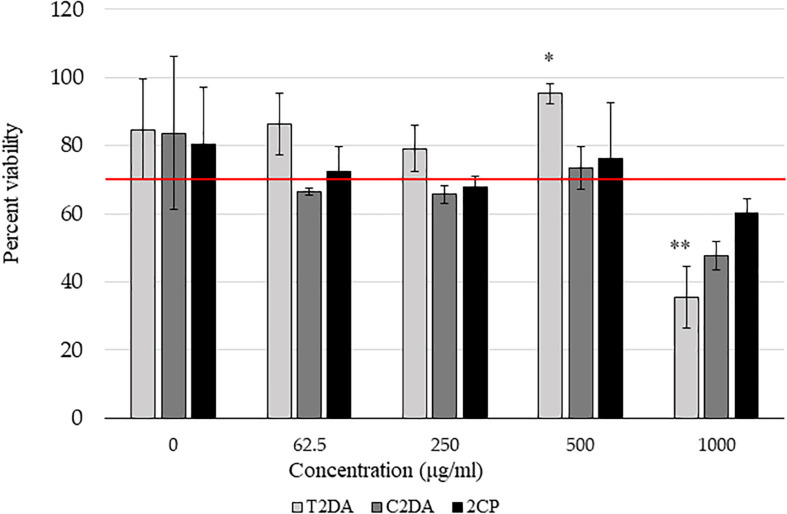
NIH-3T3 cytocompatibility assay results showing the percentage of viable cells compared to non-ethanol controls 24 h after addition of fatty acids. Asterisks represent significant differences between the other groups (^∗^*p* < 0.05, ^∗∗^*p* < 0.01), detected by one-way ANOVA with the Holm–Sidak *post-hoc* tests. Data represent mean ± standard deviation.

2CP at concentrations at 500 μg/ml in 2.5% ethanol, as well as lower concentrations, promoted fibroblast cell viability above 70% compared to non-ethanol controls, in accordance with the ISO 109935 Biological Evaluations of Medical Devices standard when evaluating biomaterials against fibroblasts. There were no statistically significant differences in cytotoxicity between 2CP and C2DA for any concentration studied; T2DA at concentrations of 500 μg/ml had slightly more viable cells remaining compared to C2DA and 2CP but significantly lower cell viability at concentrations of 1,000 μg/ml (Figure 2).

### Stability of 2CP and C2DA

**FIGURE 3:**
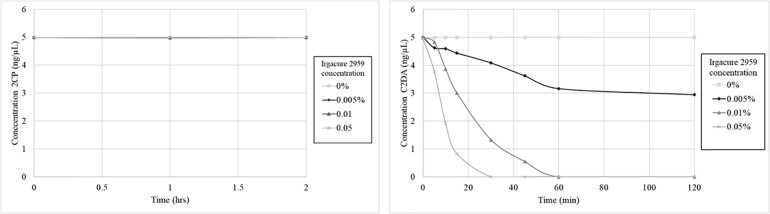
Graphs show 2CP (left) and C2DA (right) short term solution stability over time (4 h total, 2 h shown) and concentration dependence of DSF stability when challenged with UV and the photoinitiator Irgacure.

The stability of C2DA and 2CP was determined during short-term exposure to the UV-activated photoinitiator Irgacure 2959. This system was used to mimic both oxidative degradation and UV-induced isomerization of the *cis*-alkene in C2DA. C2DA was completely consumed by the combination of UV and initiator (0.05% Irgacure, half-life ∼20 min, complete degradation by 60 min, 0.1% Irgacure half-life ∼10 min, complete degradation by 30 min) (Figure 3). C2DA showed no degradation to UV alone out to 4 h of exposure. In contrast, 2CP showed no degradation to either UV alone or UV plus initiator out to 4 h.

## Discussion

A synthetic strategy for reliable synthesis of C2DA, T2DA, and 2CP is demonstrated using a combination of Lindlar reduction, Jones oxidation, and cyclopropanation to make all three targets. Our work demonstrates increased activity against *S. aureus* with a cyclopropyl isostere replacing the double bond to attain structural similarity. Chemical mimics or analogs of fatty acids like 2CP may be used in therapeutics for prevention or treatment of biofilm-based infections (Davies and Marques, 2009). Delivered locally or applied topically, 2CP may inhibit formation of biofilms or increase eradication of biofilms on implants or tissue, especially when combined with antibiotic administration. The cyclopropanated analogs have reduced isomerization and degradation when exposed to UV in the presence of a photoinitiator and thus may have better shelf stability. Further, as 2CP is an analog of natural diffusible signaling factor molecules, presence of this fatty acid is not likely to drive bacterial resistance due to selective pressure, although this has not been evaluated in the current study.

The tolerance of biofilms to antimicrobials and persistence of biofilm infections can cause unique challenges for the treatment of infections. Numerous methods are adopted for the disruption, inhibition, and eradication of biofilms. 2CP showed dispersal of biofilms, although at higher concentrations than were observed in studies of C2DA by Davies and Marques (2009). Potential reasons for these discrepancies may include use of different commercial suppliers or synthetic routes. C2DA isolated from biological spent media may also be enantiopure, which may explain the higher activity of chloroform-extracted media used in studies by Davies and Marques (2009). We are currently working to separate the two 2CP enantiomers *via* conversion to diastereomeric analogs using chiral alcohols, although this work was done using the racemic mixture. Furthermore, methods to quantify biofilms vary greatly between previous studies, which may further explain differences seen in effective concentrations. Rahmani-Badi et al. observed two- to three-fold increases in the number of planktonic cells of mixed species biofilms after treatment with 310 nM C2DA in models of catheter infection and dental plaque removal (Rahmani-Badi et al., 2014, 2015a). The activity of the *trans*-isomer against *P. aeruginosa* is consistent with studies by Davies et al. where T2DA showed dispersal activity in the concentration range reported here, although the C2DA analog dispersed biofilms at a much lower concentration (Davies and Marques, 2009). T2DA is also known as a Streptococcal DSF (SDSF), produced by *Streptococcus mutans* with inhibitory effects on fungal hyphae (Vílchez et al., 2010).

DSF molecules are known to have mechanisms of action that differ between strains at least in the site of action, external or internal to the cell. In *Xanthomonas* and *Pseudomonas*, DSF binds to specific transmembrane protein receptors and activates genetic transcription (Van Houdt and Michiels, 2010; Rahmani-Badi et al., 2015b; Ryan et al., 2015; Suppiger et al., 2016). In *Burkholderia cenocepacia*, DSF diffuse across the membrane to bind to cytosolic proteins to activate transcriptional changes (Suppiger et al., 2016). Cui et al. (2019) developed a DSF analog that was capable of interfering with *Burkholderia cenocepacia* quorum sensing and virulence, demonstrating its potential as a novel antibacterial agent. Similarly, Huedo et al. (2019) developed DSF that could interfere with quorum sensing of both *Stenotrophomonas maltophilia* and *Burkholderia cepacia*. Su et al. (2011) synthesized 4,5-disubstituted-2-aminoimidazole-triazole conjugates that inhibited *S. aureus* at micromolar concentrations and *Acinetobacter baumannii* at slightly higher concentrations, with dispersal and inhibition activity two orders of magnitude greater than C2DA. While mechanisms of action for DSF are not entirely elucidated, our cyclopropyl analog has a similar structure to C2DA and other DSF molecules and thus may have similar biological interactions with receptors and cell membranes. In one dispersal study, Rahmani-Badi et al. (2015b) have identified protein transcription changes occurring in *P. aeruginosa* upon stimulation with C2DA, identifying a cluster of genes implicated in sensing and responding to fatty-acid signals, including regulation of pathogenicity factor (rpf) proteins. It has also been shown that the histidine kinase PA1396 in *P. aeruginosa* is a major factor in DSF function; certain DSF analogs are able to block the ability of PA1396 to autophosphorylate, which subsequently reduces biofilm formation and antibiotic tolerance both *in vitro* and *in vivo* (An et al., 2019). Previous research has also demonstrated that C2DA increased membrane permeability of *P. aeruginosa* and *S. aureus* (Masters et al., 2016), which may occur by substitution of membrane phospholipids by fatty acid or by stimulating proton-motive force at the membrane (Meylan et al., 2017). Compared to enzymatic dispersal agents such as enzymatic hydrolases that degrade the EPS matrix (Fleming et al., 2017), 2-decenoic acid analogs appear to act at the cellular level and do not directly affect EPS (Marques et al., 2015).

In order to have clinical relevance as a prophylactic agent, biofilm inhibitors should also prevent planktonic bacterial growth and attachment to surfaces. Previous work has shown that C2DA has additive or synergistic growth- and biofilm-inhibitory effects with many common antibiotics including vancomycin, linezolid, tetracycline, amikacin, and ciprofloxacin (Marques et al., 2015; Masters et al., 2016; Harris et al., 2017). The present study indicates that 2CP maintains additive or synergistic effects with these antibiotics against both gram-positive and gram-negative pathogens. Similar results have been observed with drugs that target quorum sensing pathways, thereby keeping bacteria in the more susceptible planktonic state (Christensen et al., 2012; Yang et al., 2012; Roy et al., 2013). C2DA, T2DA, and the synthetic analog 2CP may be advantageous compared to antibiotic drugs because they are a natural part of the biofilm regulatory process, thereby reducing the risk of pathogens acquiring resistance (Rahmani-Badi et al., 2015b). While it has not been determined whether combining 2-decenoic acids or their analogs decrease or increase the likelihood of antimicrobial resistance, combinatorial effects should improve the efficacy of prophylactic antibiotic therapy by preventing bacterial attachment and reducing the amount of drug needed for bacterial clearance. The tobramycin–2CP combination is especially promising due to the low concentration of 2CP needed for inhibition or eradication of both bacterial strains, as well as the prevalence of tobramycin in many current local drug delivery systems.

Use of biofilm dispersal agents in the absence of antimicrobials could lead to negative effects on the host, including seeding of further infection sites and septicemia (Fleming and Rumbaugh, 2018). The present study found that 2CP increased susceptibility of established biofilms to eradication by antimicrobials, which may be due to its activity in stimulating dormant bacterial cell metabolism, which plays a major role in the antimicrobial tolerance of bacteria within biofilms (Del Pozo and Patel, 2007). Although the current study did not specifically select for persister cell populations, studies of C2DA have shown that it can revert persister cell status by increasing the respiratory activity of the cells (Marques et al., 2014). Studies by Marques et al. also demonstrated that in contrast with other metabolites such as sugars that awaken persister cells (Allison et al., 2011), C2DA is not used by bacteria as a carbon source for growth (Marques et al., 2014). The 2CP analog would not be expected to be utilized as a carbon source and further would have increased structural stability that would improve long-term storage and sterilization for healthcare applications. Biomaterial systems for co-delivery of antimicrobials and fatty-acid biofilm-disrupting agents have included direct application (Sepehr et al., 2014), biopolymer particles or sponges (Jennings et al., 2012), or implant coatings (Harris et al., 2017). While we did not study persister phenotypes specifically, future studies may explore the effects of this molecule on persisters.

For healthcare applications, targeting biofilm disruption without adverse effects on tissue is critical for the development of therapeutics for treating biofilm-based infection. We observed that the cyclopropyl moiety did not change the biocompatibility profile, with similar results to previous evaluations of cellular viability responses (Jennings et al., 2012; Rawson et al., 2014). A limitation of the current study is the use of a fibroblast cell line for preliminary evaluation of biocompatibility. Other studies of bone cell lines have shown that below certain concentration thresholds, fatty-acid biofilm inhibitors did not interfere with bone cell viability or mineralization (Harris et al., 2017). In an *in vivo* evaluation or orthopedic infection, a similar molecule C2DA applied to implants in phosphatidylcholine coatings was found to inhibit biofilm formation and no adverse events or increased inflammation in tissue were observed (Harris et al., 2017). Our preliminary stability evaluation indicates that 2CP is stable in environmental extremes, which may be favorable for sterilization or incorporation into controlled delivery systems. Longer-term and milder degradation experiments are currently underway to confirm enhanced the shelf stability of 2CP. Expanded preclinical evaluations, including studies on inflammatory cell effects, bone cell response, and *in vivo* infection models, are needed to fully evaluate the potential of 2CP as a clinical therapeutic. Another limitation of this study is the use of microtiter plate-based assays; while they were adequate for this pilot study, future studies will include additional biofilm assays to confirm results, in addition to experiments that may specifically identify effects of 2CP on gene expression and phenotype as well as sensing mechanisms for different bacteria. Evaluation of 2CP activity in dynamic conditions such as bioreactor culture could complement these results. While this study established preliminary efficacy against two representative strains of bacteria, future studies may also incorporate mixed cultures of different strains of bacteria as well as fungi.

We have demonstrated a successful synthetic route for cyclopropanated analogs of 2-decenoic acid, replacing the double bond in *cis*-configuration with a cyclopropyl ring. This analog had similar or improved bioactivity in biofilm dispersal, inhibition, and eradication. Additive and synergistic effects when 2CP is combined with antimicrobials may be clinically useful in applications to treat implant-associated infection, dental plaque removal, or surface decontamination. Future work will explore methods for sustained delivery, evaluate long-term stability and activity, and expand preclinical investigations to determine potential as a clinical therapeutic.

## Data Availability Statement

The raw data supporting the conclusions of this article will be made available by the authors, without undue reservation.

## Author Contributions

JJ and DB conceived this idea and determined the methodology. BR synthesized the fatty acids and performed the chemical analysis and validation. ZH, RA, and MH conducted the biofilm studies and performed the bacteriological analysis. BH performed the stability evaluation and all authors contributed to the original draft preparation. ZH, JJ, and DB reviewed and edited the final forms of this manuscript. JJ and DB acquired the funding, were administrators of this project, and supervised the other authors.

## Conflict of Interest

The authors declare that the research was conducted in the absence of any commercial or financial relationships that could be construed as a potential conflict of interest.
