# Supplementary material for: 2-Heptylcyclopropane-1-Carboxylic Acid Disperses and Inhibits Bacterial Biofilms
Source: Front Microbiol. 2021 Jun 9;12:645180. doi: 10.3389/fmicb.2021.645180 (PMC8221421; doi:10.3389/fmicb.2021.645180)
Supplement: Supplementary file 1 [file Data_Sheet_1.pdf]

**Supplementary Material**

**1. Synthesis of C2DA and 2CP**

A common synthetic approach (Scheme 1) using a single starting material was used to generate C2DA (I) via Jones oxidation and then Lindlar reduction (A and B), and 2CP (II) via Lindlar reduction, Simmons-Smith cyclopropanation, and finally Jones oxidation (B*, C*, and A*). T2DA was purchased from Cayman Chemical and was used without purification.

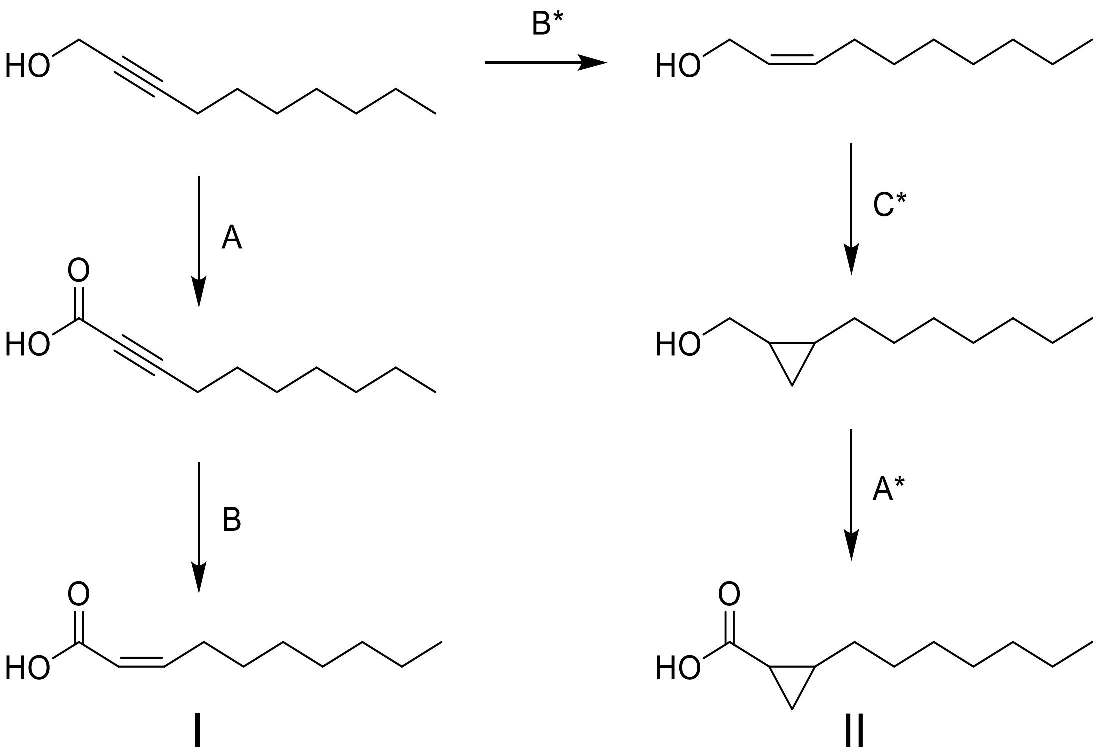


**Figure S1.** Synthesis of C2DA (I) and 2CP (II). Reagents and conditions (A and A*) Jones reagent, acetone, 0 °C to room temperature. (B and B*) Lindlar catalyst, THF, hydrogen gas. (C*) diethyl zinc, diiodomethane, tetrahydrofuran (THF), hexanes, -40 °C.

**1.1 Synthesis of C2DA (I)(A)**

A clean, dry 100mL round-bottom flask at 0 °C was charged with 1 g of 2-decyn-1-ol and 20 mL acetone, followed by the addition of freshly prepared Jones oxidation reagent (7 mL). The reaction mixture was stirred for 1 h, then extracted with 25 mL of ethyl acetate 4 times. The organic extract was washed with deionized water, brine, dried with anhydrous sodium sulfate and filtered through celite. Solvent was removed at reduced pressure to yield 2-decynoic acid which was taken to the next step without further purification. (B) Into a reaction vessel with THF, 2-decynoic acid, 70 µL quinoline, and 100 mg Lindlar's catalyst were added. The reaction mixture was flushed 3 times and then charged with 2 atm hydrogen gas before shaking in a Parr Hydrogenator for 1 h. Upon completion the mixture was filtered through celite to remove catalyst followed by flash

chromatography using a THF-hexanes gradient to yield 650 mg of cis-2-decenoic acid (C2DA, I) as a pale oil in 65% overall yield. 1H-NMR (CDCl3, 400 MHz): δ 6.34 (q, J = 11.43 Hz, 1H), 5.77 (d, J = 11.43 Hz, 1H), 2.65 (q, 2H), 1.49-1.2 (m, 10H), 0.86 (t, 3H), 13C-NMR (CDCl3, 400 MHz): δ (ppm) 172.23, 153.79, 119.05, 34.20, 31.84, 29.47, 29.30, 29.15, 22.72, 14.17, HRMS: Expected [M-H]- = 169.13 m/z, observed [M-H]- = 169.1189 m/z.

Although T2DA was purchased from Cayman Chemical, 1H NMR was collected to verify differences in both the chemical shifts and coupling constants for the vinylic protons in T2DA versus C2DA. 1H-NMR (CDCl3, 400 MHz): δ 7.09 (q, J = 15.55 Hz, 1H), 5.81 (d, J = 15.55 Hz, 1H), 2.22 (q, 2H), 1.49-1.2 (m, 10H), 0.86 (t, 3H). All C2DA generated contained no evidence of T2DA contamination as determined by 1) correct integrations (1 proton each) for C2DA vinylic protons at 6.34 and 5.77 ppm with coupling constants of 11.43 Hz, and 2) a lack of T2DA resonances at 7.09 and 5.81 ppm with coupling constants of 15.55 Hz.

**1.2 Synthesis of 2CP (II)(B*)**
A glass reaction vessel was charged 1 g of 2-decyn-1-ol, 20 ml of THF, and 70 μL of quinoline. The flask was purged with nitrogen gas for 5 minutes before adding 100 mg of Lindlar's catalyst. The reaction mixture was then purged 3 times, then pressurized with hydrogen gas to 2 atm and shaken for 1 h. Upon completion the mixture was filtered through celite and concentrated at reduced pressure to yield 900 mg of (Z)-dec-2-en-1-ol as an oil in 90% yield. 1H-NMR (CDCl3, 400 MHz): δ 5.73-5.44 (m, 2H), δ 4.18 (d, J = 5.9 Hz, 2H), δ 2.11-1.99 (m, 2H), δ 1.46-1.15 (m, 12H), δ 0.86 (t, J = 6.9 Hz, 3H). **(C*)** A round bottom flask was cooled to -40 °C with a dry ice/acetonitrile bath and put under an inert atmosphere. To this flask 900 mg (Z)-2-decenol was dissolved in 10 mL of dry dichloromethane, followed by the slow addition of 20 mL diethyl zinc (15 M in hexanes). The mixture was stirred at -40 °C for 10 minutes before adding 5.2 g of diiodomethane dropwise. The reaction mixture was allowed to gradually warm to room temperature and stirred for 24 h, diluted with hexanes, and then filtered to remove insoluble materials. The filtrate was cooled to 0 °C, and then quenched with saturated ammonium chloride solution followed by extraction with hexanes 5 times. The combined organic extracts were washed with ammonium chloride solution, water, brine, and then dried with anhydrous magnesium sulfate. The extract was filtered through celite to remove solids, and dichloromethane was removed at reduced pressure. The crude product was dissolved in acetone to remove insoluble materials, filtered and concentrated at reduced pressure to give 750 mg of crude (2-heptylcyclopropyl) methanol as a yellow oil in 83% yield. This product was taken to the next step without further purification. 1H-NMR (CDCl3, 400 MHz): δ 3.63-3.61 (1H), δ 3.58-3.54 (dd, J =8.23 Hz, 10.98 Hz, 1H), δ 1.45-1.36 (2H), δ 1.26-1.18 (11H), δ 1.13-1.04 (1H), δ 0.87 (t, 3H), δ 0.64 (td, J =4.57 Hz, 4.12 Hz, 1H), δ -0.03 (q, 1H). **(A*)** At 0 °C, 750 mg of (2-heptylcyclopropyl) methanol was dissolved in 20 mL of acetone followed by dropwise addition of 7 mL of freshly prepared Jones oxidation reagent. The reaction mixture was stirred for 1 h, diluted with water, and extracted with hexanes 5 times. The combined extracts were washed with water, brine, dried with anhydrous magnesium sulfate, and concentrated under reduced pressure. The crude product was purified via flash chromatography on silica gel using an ethyl acetate-hexanes gradient to yield 500 mg of 2CP as a pale yellow oil in 55% yield (50% overall yield). 1H-NMR (400 MHz, CDCl3) δ 1.64 (d, J = 7.8 Hz, 1H), 1.52 (d, J = 7.7 Hz, 2H), 1.19-1.43 (11H), 1.06 (dd, J = 16.3, 4.5 Hz, 1H), 0.95 (dd, J = 9.9, 7.4 Hz, 1H), 0.88-0.85 (m, 3H)

13C-NMR (101 MHz, CDCl3) δ 180.0, 31.9, 29.6, 29.3, 29.3, 27.0, 23.3, 22.7, 18.1, 14.5, 14.21
HRMS: Expected [M-H]- = 183.15 m/z, observed [M-H]- = 183.13694 m/z.

Both the synthetic approach to the synthesis of 2CP (Lindlar reduction to the cis alkene, followed by Simmons-Smith cyclopropanation) and the resulting $^{1}$H NMR, specifically protons B (1.64 ppm, J=7.8 Hz), C (1.52 ppm, J=7.7 Hz), K' (0.95 ppm, J=9.9 Hz), K (1.06 ppm, J= 16.33 Hz) (See Figure S2 which shows one of the two possible enantiomers of 2CP) confirm the cis-like orientation of the C2-C3 cylcopropyl group with respect to the carboxylic acid and alkyl functional groups. We are currently working to separate the two 2CP enantiomers via conversion to diastereomeric analogs using chiral alcohols. But this work was done using the racemic mixture.

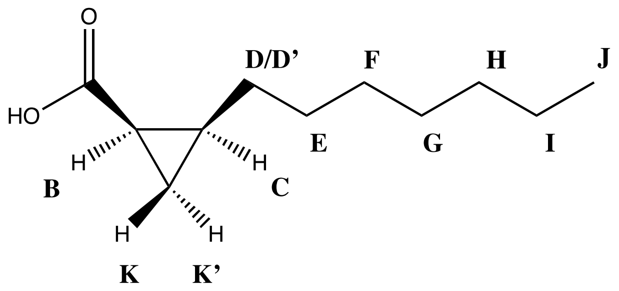


Figure S2. Structure of one enantiomer of 2CP (2R, 3S) with proton positions labeled.

Utilizing the J-coupling values from the $^{1}$HNMR shown in Figure S3 it can be determined that the 2CP that has been synthesized is "cis-like" in conformation with protons B, C, and K' having J-coupling values of 7.8, 7.7, and 9.8 respectively which are all within the "cis-like" range between 5-14 Hz. The K proton has a J-Coupling value of 16.3 which falls into the "trans-like" range between 11-19 Hz. Utilizing these coupling constants we can confirm we have in some mixture of (1R,2S)-2CP and (1S,2R)-2CP. With these proton shifts, integrations and coupling constants aligning to the previous work of Widder et al. the cis like product can be confirmed [1].

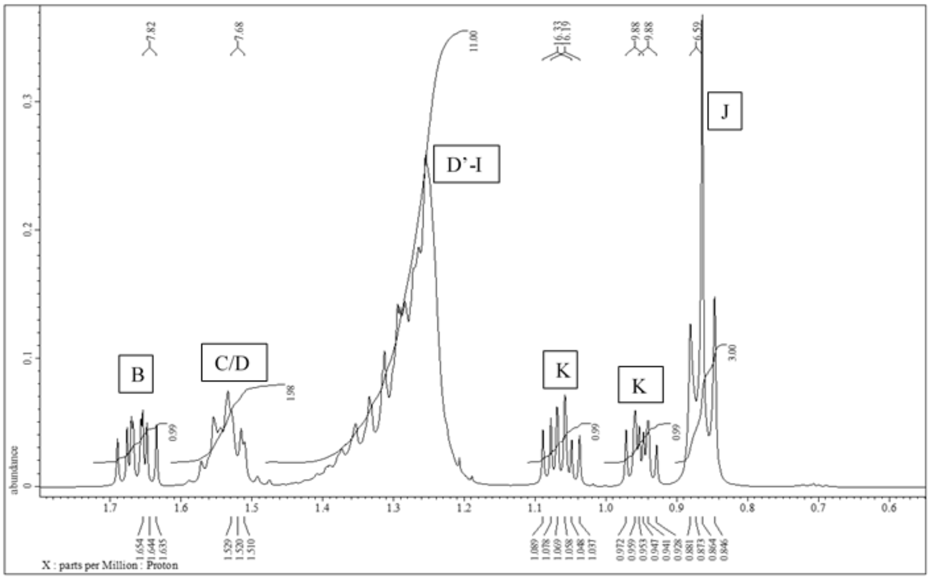


**Figure S3.** $^{1}$HNMR spectra showing J-coupling values for protons of 2CP labeled in Figure S2.

[1] S. Widder, J. Looft, A. Van Der Kolk, T. Vössing, W. Pickenhagen, and B. Kohlenberg, 2-heptylcyclopropyl-1-carboxylic acid, Google Patents, 2010.
